# Therapeutic efficacy of drilling drainage combined with intraoperative middle meningeal artery occlusion in the management of chronic subdural hematoma: a clinical study

**DOI:** 10.1007/s10143-024-02501-1

**Published:** 2024-06-25

**Authors:** Tao Sun, Dongqi Shao, Jian Li, Decai Xu, Tao Zhang, Lei Li, Wenjie Sun, Caihong Zhang, Xinjie Wen, Haonan Chen, Renhao Zhang, Zhiquan Jiang

**Affiliations:** 1 School of Continuing Education, Anhui Medical University, Hefei, China; 2 Department of Neurosurgery, The First Affiliated Hospital of Bengbu Medical University, Bengbu, China

**Keywords:** Chronic subdural hematoma (CSDH), Cranial drilling and drainage, Middle meningeal artery (MMA), Pathogenesis, Treatment

## Abstract

**Background:**

The bone holes in the skull during surgical drainage were accurately located at the site of the MMA. The MMA was severed, and the hematoma was removed intraoperatively; furthermore, surgical drainage removed the pathogenic factors of CSDH. This study aimed to describe and compare the results of the new treatment with those of traditional surgical drainage, and to investigate the relevance of this approach.

**Methods:**

From December 2021 to June 2023, 72 patients were randomly assigned to the observation group and the control group. The control group was treated with traditional surgical drainage, while the observation group was treated with DSA imaging to accurately locate the bone holes drilled in the skull on the MMA trunk before traditional surgical drainage. The MMA trunk was severed during the surgical drainage of the hematoma. The recurrence rate, time of indwelling drainage tube, complications, mRS, and other indicators of the two groups were compared, and the changes of cytokine components and imaging characteristics of the patients were collected and analyzed.

**Results:**

Overall, 27 patients with 29-side hematoma in the observation group and 45 patients with 48-side hematoma in the control group were included in the study. The recurrence rate was 0/29 in the observation group and 4/48 in the control group, indicating that the recurrence rate in the observation group was lower than in the control group (*P* = .048). The mean indwelling time of the drainage tube in the observation group was 2.04 ± 0.61 days, and that in the control group was 2.48 ± 0.61 days. The indwelling time of the drainage tube in the observation group was shorter than in the control group (*P* = .003). No surgical complications were observed in the observation group or the control group. The differences in mRS scores before and after operation between the observation group and the control group were statistically significant (*P* < .001). The concentrations of cytokine IL6/IL8/IL10/VEGF in the hematoma fluid of the observation and control groups were significantly higher than those in venous blood (*P* < .001). After intraoperative irrigation and drainage, the concentrations of cytokines (IL6/IL8/IL10/VEGF) in the subdural hematoma fluid were significantly lower than they were preoperatively. In the observation group, the number of MMA on the hematoma side (11/29) before STA development was higher than that on the non-hematoma side (1/25), and the difference was statistically significant (*P* = .003).

**Conclusion:**

In patients with CSDH, accurately locating the MMA during surgical trepanation and drainage, severing the MMA during drainage, and properly draining the hematoma, can reduce the recurrence rate and retention time of drainage tubes, thereby significantly improving the postoperative mRS Score without increasing surgical complications.

## Introduction

Chronic subdural hematoma (CSDH) is one of the most common diseases in the department of neurosurgery [1, 2]. According to the literature, the annual incidence of CSDH is 13.5 per 100,000 people [3]; however, its annual incidence can be as high as 80.1 per 100,000 people among individuals over 65 years of age, and even 127.1 per 100,000 people in those over 80 years of age [4]. It is anticipated that by 2030, CSDH will become the most common cranial neurosurgical pathology affecting adults [3, 5]. The acceleration of social life, the exacerbation of population aging, and the widespread utilization of anticoagulant and antiplatelet aggregation medications have contributed to a progressive rise in the annual incidence and mortality rate of CSDH [6, 7]. It is expected that in the next 10 years, the incidence of CSDH will double [8]. Without proper treatment, CSDH can lead to increased intracranial pressure, neurological dysfunction, psychiatric symptoms [9–11], and other symptoms, such as headache, mental decline, hemiplegia, coma, and even death [12]. Such adverse effects and outcomes significantly affect people’s quality of life and threaten human health [13].

Currently, the treatment modalities for CSDH are classified into drug therapy and surgical treatment. Due to uncertain efficacy, long medication time, side effects, and the inability to rapidly remove the space-occupying effect of intracranial hematoma, drug therapy can only be used as an adjunctive treatment method for CSDH [14]. Currently, surgery is the main treatment modality for CSDH. Cranial drilling and drainage can quickly eliminate the space-occupying effect and reduce intracranial pressure, leading to good prognoses in more than 80% of patients; therefore, it has become the preferred, most widely used, and classic surgical method for CSDH worldwide [15–17]. Although cranial drilling and drainage has the characteristics of being a simple operation with minimal invasiveness [18] that does not require complex surgical conditions and equipment and has a good clinical prognosis, cranial drilling and drainage has a high recurrence rate. Reports show that the recurrence rate of hematomas after surgical intervention using traditional methods for CSDH is high—up to 37% [19–23]. Therefore, there is an urgent need to find a treatment method that can reduce the postoperative recurrence rate of CSDH.

To cure a disease, a good understanding of the disease and its pathogenesis is required. Based on a large number of clinical observations and experimental studies, it is believed that the pathophysiological mechanism of CSDH has the following main aspects: inflammation, angiogenesis, coagulation disorders, and hyperfibrinolysis [24]. External drainage via drilling can lead to the removal of pro-inflammatory factors and abnormal coagulation substances from the hematoma cavity; however, it cannot remove angiogenic factors. Relevant studies have indicated that in a certain sense, the blood supply to CSDH is obtained from the dural branch of the middle meningeal artery (MMA) [25], leading to repeated and chronic leakage, which leads to the progression of the disease. If the blood supply to a CSDH—from the MMA—is blocked and the cycle of repeated and chronic leakage is eliminated, the accumulated blood in the subdural space can be gradually absorbed over time. Currently, MMA embolization is a treatment modality for CSDH that employs the abovementioned mechanism [26]. If one method can simultaneously remove several etiologies of CSDH, it is theoretically an ideal treatment method. In this study, the MMA was identified using DSA, and a drill hole was made on the skull to expose the dural MMA during cranial drilling for drainage. The MMA was severed during drainage to remove inflammatory factors and abnormal coagulation substances, and to block angiogenic factors. Other related clinical observations and studies were also conducted.

## Methods

### Study population and patient selection

Patients with CSDH who required surgery at the Department of Neurosurgery of the First Affiliated Hospital of Bengbu Medical University between December 2021 and June 2023 were selected. The inclusion criteria were as follows: patients with CSDH confirmed by head imaging examination (CT or MR); patients aged over 30 years; patients with greater than 5 mm displacement of midline structures or a hematoma thickness greater than 10 mm [25]; and patients with specific clinical symptoms, including symptoms of chronic intracranial hypertension, such as headache, nausea, vomiting, mental and intellectual symptoms such as mental retardation, mental abnormality, memory decline, and focal symptoms such as hemiplegia and aphasia. The exclusion criteria were as follows: patients with capsule thickening or calcification requiring large bone flap craniotomy for hematoma capsule resection; patients who were reluctant to undergo surgery and required conservative treatment; patients with cardiopulmonary, liver, kidney, and other organ dysfunction who could not tolerate surgery; and patients with severe trauma in other parts.

### Intervention and grouping

**Fig. 1:**
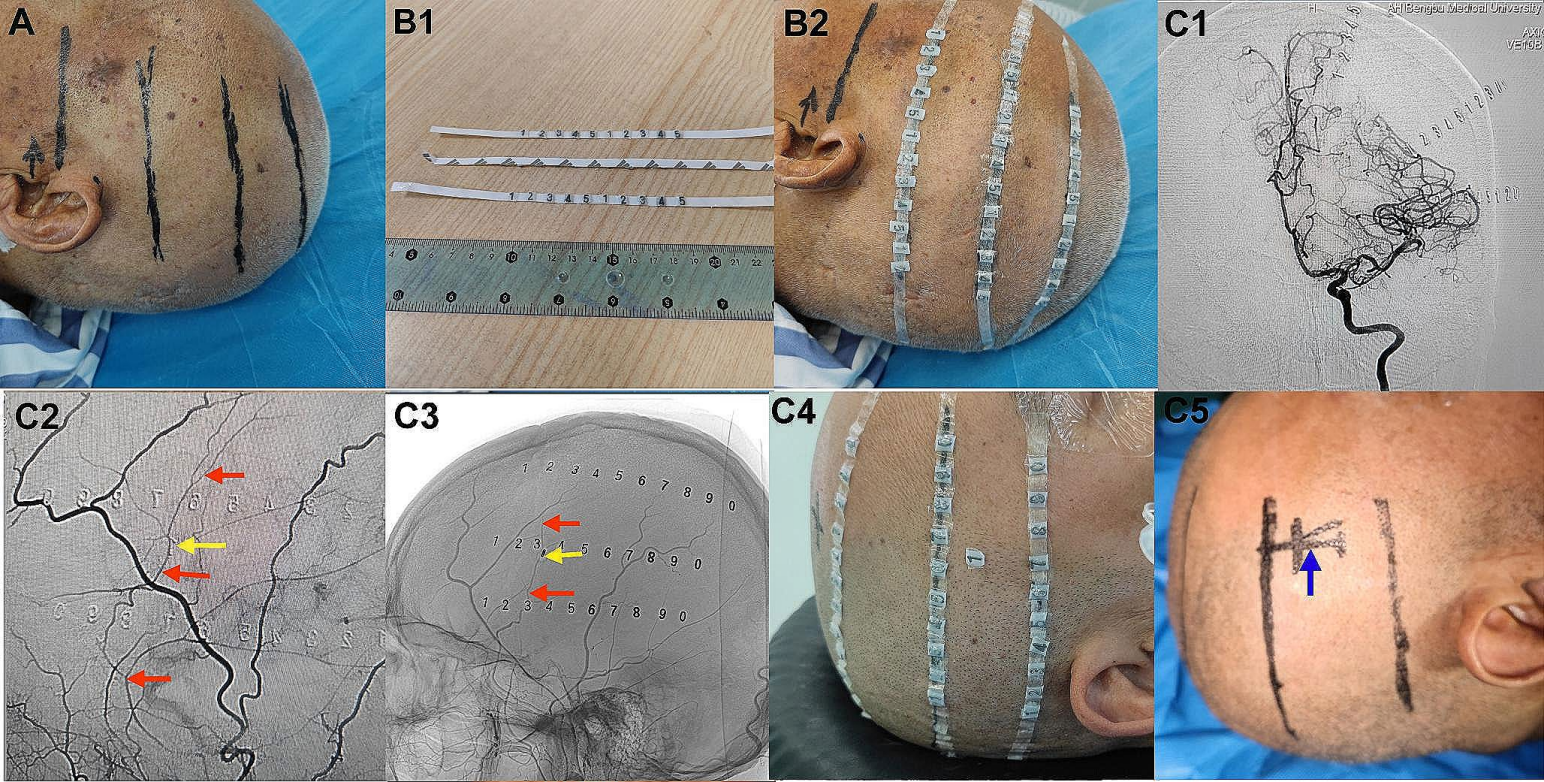
1 A The location of the hematoma is determined according to the head image before surgery, and the lowest, highest, and thickest levels of the hematoma are marked. 1B1 A double-sided tape is affixed with type 1 cm apart. 1B2 Paste the double-sided tape affixed with type to the lowest and highest levels of the head hematoma and the thickest level of the hematoma. 1C1 Coronal imaging showed that the Intracranial blood vessels on the side of the hematoma was compressed and displaced by the hematoma. The optimal blocking point of the MMA could be adjusted according to the shape of the hematoma and the positioning type in the middle. 1C2 Lateral imaging of external carotid artery, the Type 1 (yellow arrow) marks the MMA (red arrow). 1C3 Lateral imaging of external carotid artery, positioning needle (yellow arrow) marks the MMA (red arrow). 1C4 Apply Type 1 to the corresponding scalp surface according to the location of the MMA using contrast. 1C5 Draw the incision line on the scalp according to imaging positioning (blue arrow).

**Fig. 2:**
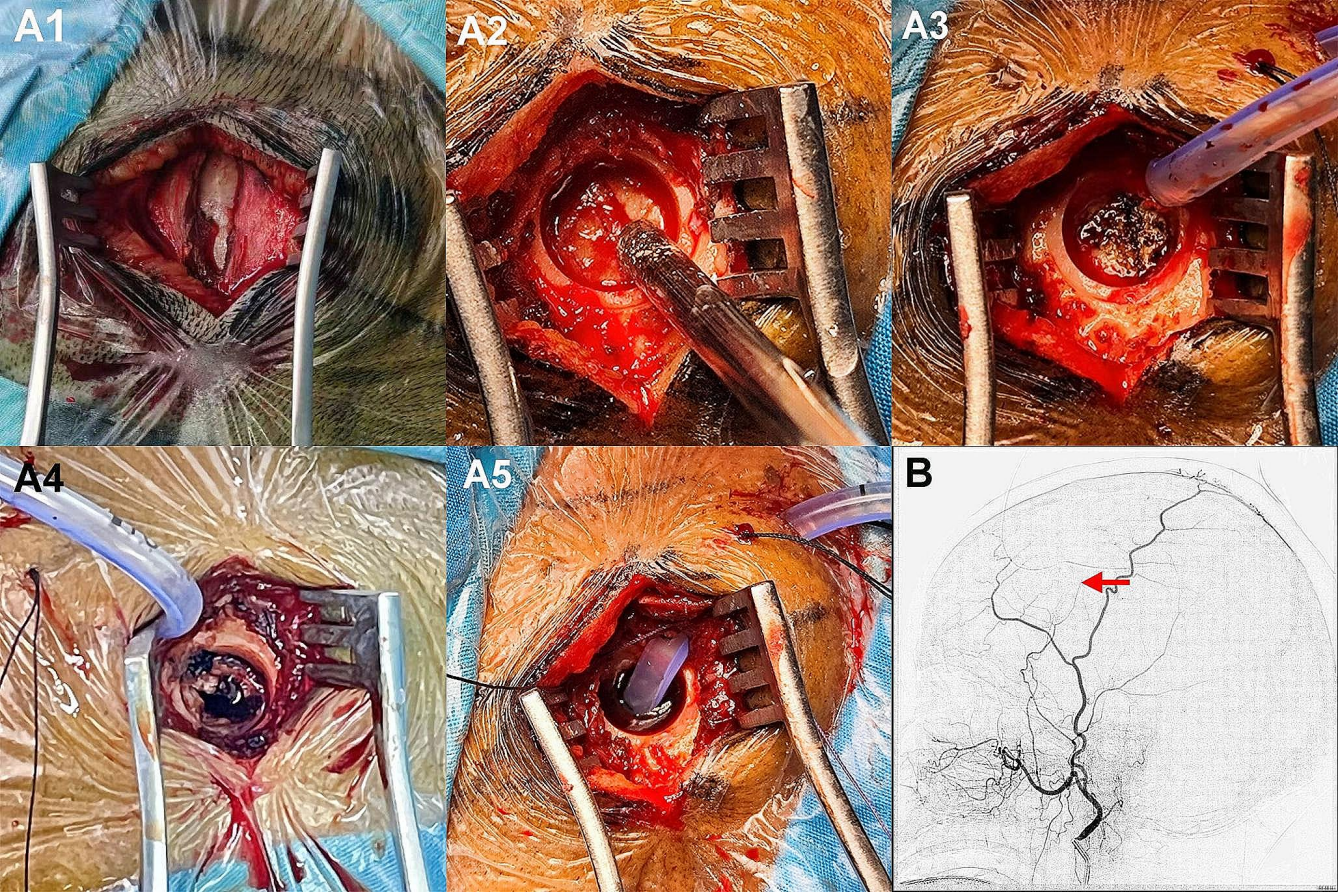
2A1 Divide the temporal muscle along the temporal muscle fibers after making a scalp incision. 2A2 Skull drilling to accurately locate the MMA. 2A3 Electrocoagulation of the MMA. 2A4 Incising the dura matter and the MMA. 2A5 A drainage tube is placed in the subdural space for drainage. 2B After the operation, external carotid arteriography is performed on the diseased side to examine the extent of MMA damage (red arrow shows the severed MMA)

The patients were randomly divided into the observation group and the control group. All patients and at least one family member signed informed consent forms. All antiplatelet medications were discontinued preoperatively. General anesthesia was used during the operation, and real-time monitoring of blood oxygen saturation, heart rate, electrocardiogram waveform, and blood pressure was performed. The site of the hematoma was identified using preoperative cranial images in the observation group. The lowest, highest, and thickest horizontal planes of the hematoma were also marked [Fig. 1A]. The corresponding layers were double-sided and glued with markings [Fig. 1B1B2]. DSA of the common (or internal) carotid artery on the healthy side, the healthy side of the external carotid artery, the vertebral artery, the common (or internal) carotid artery and the external carotid artery on the side of hematoma were performed through the femoral artery. Using angiographic images of the internal carotid artery on the side of the lesion, which showed the deformation and displacement of the vessels that were compressed by the hematoma along with the horizontal plane of the preoperative site of the hematoma, the puncture plane slightly below the thickest horizontal plane of the hematoma was selected. External carotid angiography on the lesion side combined with the metal marker table was also used to locate the path of the main trunk of the MMA. The puncture point was located at the intersection of the puncture plane and the main trunk of the MMA [Fig. 1C1-C5]. If it was a bilateral lesion, the left and right puncture points were respectively located and marked. After angiography, the hematoma cavity was drilled for drainage. A 25-mm-long incision was made on the scalp, the temporal muscle was separated along its fibers, and the skull was exposed. A small hole with a diameter of approximately 8 mm was drilled at the puncture point, and the MMA was identified. After electrocoagulation, the dura and MMA were cut open, and the hematoma fluid was collected. A drainage tube was placed in the hematoma cavity, and the drainage tube in the head was placed in the largest and thickest part of the hematoma [Fig. 2A1-A5]. The hematoma cavity was also rinsed repeatedly with normal saline. DSA imaging on the lesion side was performed postoperatively to determine the extent of damage to the MMA [Fig. 2B]. In the control group, the selected puncture point was located at the thickest part of the hematoma; the other components of the operations were the same as those of the observation group. The patients in both groups underwent cranial CT postoperatively. If the drainage tube was located in the hematoma cavity but the drainage was not inadequate, and there was still the hematoma, 40,000 units of urokinase were injected into the hematoma cavity while observing strict asepsis, and the drainage was opened after 1 h.

**Fig. 3:**
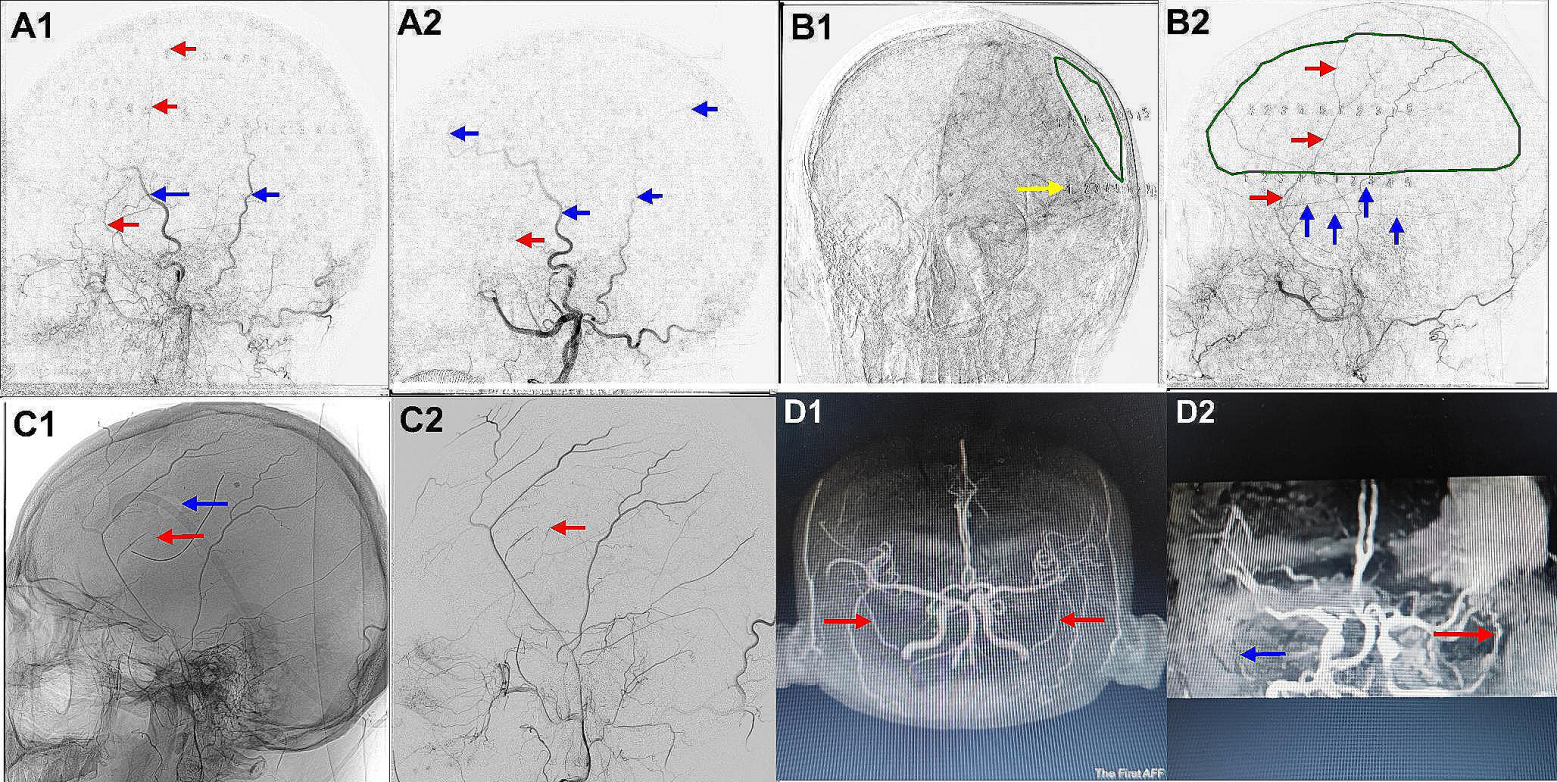
3A1 Preoperative external carotid arteriograph on the hematoma side shows the MMA (red arrow) appears before the STA (blue arrow).3A2 Postoperative external carotid arteriograph on the hematoma side shows the STA (blue arrow) appears before the MMA (red arrow). 3B1 Internal carotid artery arteriograph of the side with the hematoma shows the hematoma (green circle) with its lower boundary at the bottom (yellow arrow). 3B2 External carotid artery arteriograph on the hematoma side of the same patient shows that the hematoma (green circle) is mainly distributed in the anterior branch of the MMA (red arrow), and the posterior branch of the MMA (blue arrow) is largely located at the lower boundary of the hematoma. 3C1 Lateral imaging of external carotid artery shows that the position of the MMA stump (red arrow) is lower than that of the bone foramen (blue arrow). 3C2 Lateral cut film of the external carotid artery in the same patient shows the location of the MMA stump (red arrow). 3D1 MRA in a healthy person shows the symmetry of the bilateral MMA (red arrow). 3D2 MRA of a patient with CSDH, the MMA on the hematoma side (red arrow) is thickened and more pronounced than that on the healthy side (blue arrow)

### Outcome measures

All patients were followed up for 5 months postoperatively (because the median time for hematoma absorption after MMA blockade was 161 days, and most patients relapsed within 5 months) [27, 28]. Relevant data were collected, including pre- and post-operative imaging data: CT hematoma density (high density, isodensity, low density, and mixed density) or MR signal (uniform signal, mixed signal); site of hematoma; hematoma thickness (measured the width of the thickest part of the hematoma on the affected side, the mean value of both sides of the bilateral hematoma), etc. Surgical complications (infection, rebleeding, cerebrospinal fluid leakage, brain contusion and laceration, epilepsy, and poor scalp wound healing) were compared between the two groups. Changes in mRS functional scores pre- and postoperatively, the duration the indwelling drainage tube was in situ, and the levels of interleukin 6/8/10 and VEGF in the hematoma fluid intraoperatively and before extubation were recorded. The recurrence rate of hematoma within 5 months after surgery. The patient underwent a follow-up CT or MR scan of the head on the first day after surgery, before extubation, before discharge, at 1, 3, and 5 months after surgery, and if new symptoms developed. Hematoma recurrence was defined as the occurrence of a hematoma on the operated side within 5 months that required a second surgery due to its space-occupying effect.

### Statistical analysis

The data collected were analyzed using SPSS version 21.0 (IBM Corp., Armonk, NY, USA). The normal distribution and variance homogeneity tests were performed on the measurement data. The normal distribution measurement data were expressed as mean ± standard deviation, and the skewed measurement data were expressed as median ± interquartile range. The paired or independent sample t test was used for the comparison of the two groups of means according to the actual situation. The Wilcoxon signed-rank test was used for paired data with a non-normal distribution, and the Wilcoxon rank-sum test (Mann-Whitney U test) was used for non-normal unpaired data. *P* values of < 0.05 were considered to indicate statistical significance.

## Results

### Patients’ characteristics

72 patients (63 male (87.5%) and 9 female (12.5%) patients; age 37–88 years; mean age, 71.54 ± 11.49 years) participated in this study. Overall, 27 patients were in the observation group, while 45 patients were in the control group. A total of 33 patients had left sided hematomas, 34 had right-sided hematomas, and five had bilateral hematomas. Regarding hematoma density, CT images showed low-density hematomas in 37 cases, isodense hematomas in 8 cases, high-density hematomas in 10 cases, and mixed density hematomas in 22 cases. The maximum thickness of the hematoma was between 10 mm and 20 mm on 53 sides, between 20 mm and 30 mm on 21 sides, and greater than 30 mm on three sides. Additionally, CT showed that midline structures were shifted by less than 5 mm in five cases, between 5 mm and 10 mm in 45 cases, between 10 mm and 20 mm in 20 cases, and greater than 20 mm in two cases. Common symptoms upon presentation included headache in 28 cases, dizziness in 13 cases, mental abnormality in nine cases, aphasia in six cases, limb numbness in 31 cases, a decline in limb muscle strength in 58 cases, and epilepsy in one case. There were 46 cases of hypertension and 17 cases of diabetes mellitus. Before admission, 20 patients were on oral aspirin therapy and one patient was on oral clopidogrel therapy; however, no patient was on aspirin and clopidogrel simultaneously. The patients’ coagulation indices, including platelet count, PT, APTT, D-dimer, etc., were evaluated preoperatively and are shown along with the patients’ baseline characteristics in Table 1.

**Table 1:** Preoperative characteristics of the patients (No./total No.(%))

Variable	Observation Group(*N* = 27)	Control Group(*N* = 45)
Age, mean ± SD	71.8 ± 11.3	72.3 ± 10.7
Women	2/27(7.4%)	7/45(15.5)
Men	25/27(92.5)	38/45(84.4)
Laterality		
Left only	11/27(40.7%)	22/45(48.9%)
Right only	14/27(51.9%)	20/45(44.4%)
Bilateral	2/27(7.4%)	3/45(6.7%)
CT image		
Hypodense	12/29(41.4%)	25/48(52.1%)
Isodense	4/29(13.8%)	4/48(8.3%)
Hyperdense	3/29(10.3%)	7/48(14.6%)
Mixed density	10/29(34.5%)	12/48(25.0%)
MR image		
Uniform signal	4/10(40.0%)	4/12(33.3%)
Mixed signal	6/10(60.0%)	8/12(66.7%)
Maximal hematoma width, mm		
10 mm ≤ 53 sides<20 mm	20/29(69.0%)	33/48(68.8%)
20 mm ≤ 21 sides<30 mm	8/29(27.6%)	13/48(27.1%)
3 sides ≥ 30 mm	1/29(3.4%)	2/48(4.2%)
Midline shift, mm		
5 cases<5 mm	2/27(7.4%)	3/45(6.7%)
5 mm ≤ 45 cases<10 mm	17/27(63.0%)	28/45(62.2%)
10 mm ≤ 20 cases<20 mm	8/27(29.7%)	12/45(26.7%)
2 cases ≥ 20 mm	0/27(0%)	2/45(4.4%)
Symptoms at admission		
Headache	11/27(40.0%)	17/45(37.8%)
Dizziness	5/27(18.5%)	7/45(15.6%)
Altered mental status	3/27(11.1%)	6/45(13.3%)
Dysphasia	2/27(7.4%)	4/45(8.9%)
Limb paraesthesia	12/27(44.4%)	19/45(42.2%)
Limb muscle strength decreased	21/27(77.8%)	37/45(82.2%)
Seizure	0/27(0%)	1/45(2.2%)
Comorbidities		
Hypertension	18/27(66.7%)	28/45(62.2%)
Diabetes mellitus	7/27(25.9%)	10/45(22.2%)
Antiplatelet medication		
Aspirin	7/27(25.9%)	13/45(28.9%)
Clopidogrel	1/27(3.7%)	0/45(0%)
Aspirin + Clopidogrel	0/27(0%)	0/45(0%)
Preoperative venous blood test indicators, mean ± SD		
Platelet count (×109/L)	174.01 ± 69.43	183.38 ± 58.47
prothrombin time (sec)	10.98 ± 2.56	12.75 ± 12.55
Activated partial thromboplastin time (sec)	26.42 ± 6.27	26.68 ± 4.69
D-Dimer (mg/L)	1.42 ± 2.62	1.12 ± 1.91
CRP (mg/L)	4.09 ± 4.42	5.49 ± 8.05

### Surgical conditions

Drainage tubes were successfully inserted into the hematoma cavities of patients in both the observation and control groups, and the hematoma fluid was successfully drained. Patients in both the observation and control groups were administered urokinase injections postoperatively on nine sides and on 17 sides of the hematoma, respectively. Patients who were administered urokinase injections did not report any adverse effects, and subsequent DSA examinations revealed intracranial vascular stenosis exceeding 50% in three patients in the observation group. In patients who underwent early DSA, a lead was used to locate the MMA. Due to the thickness of the scalp, temporal muscle, and skull, no vertical lead could be used to locate the MMA during angiography, resulting in the failure to accurately locate the MMA during the operation after marking the site of the incision. Two patients dropped out of the observation group and the control group. In patients in whom the MMA was located during a later DSA, the problems of non-vertical angiography and the lead were resolved, and the disinfected needle localization method was used to accurately locate the MMA. The MMA was accurately located below the site of the bone hole drilling. DSA of the internal carotid artery in 27 patients with hematomas on 29 sides showed that the vessels of the cerebral cortex were compressed and deformed by the hematoma, leading to the formation of a “fusiform” image along with the skull. The upper and lower boundaries of the “fusiform” hematoma image were basically consistent with the position of the lead preoperatively [see Fig. 1C1]. To safely make a thick puncture point as far as possible below the hematoma, the optimal puncture plane in several cases was adjusted according to the position of the “fusiform” hematoma image.

### Clinical results

#### IL/VEGF expression level

Figure 5 shows the levels of IL6/8/10 and VEGF in preoperative venous blood, hematoma fluid without saline washing intraoperatively, and the drainage tube before extubation in the control and observation groups. There were statistically significant differences between the concentrations of IL6/8/10 and VEGF in preoperative venous blood and intraoperative hematoma fluid without saline washing in the observation group and the control group. There were no statistically significant differences in the concentrations of IL6/8/10 and VEGF in the hematoma fluid obtained without saline washing and in hematoma fluid in the drainage tube before extubation between the observation group and the control group. There were statistically significant differences in the concentrations of IL6/10 and VEGF in the hematoma fluid obtained without saline washing and in the hematoma fluid in the drainage tube before extubation in the observation group; however, there was no statistically significant difference in the levels of IL8. On the other hand, statistically significant differences were observed in the concentrations of IL6/8 and VEGF in the hematoma fluid obtained without saline washing and in hematoma fluid in the drainage tube before extubation in the control group; however, there was no statistically significant difference in the levels of IL10. During the operation, when the dura mater on the lesion side was incised, a small amount of dura mater was randomly left in the observation group and the control group for VEGF immunohistochemical detection. The positive rate in the observation group was 85.7% (6 cases), and the positive rate in the control group was 57.1% (4 cases).

**Fig. 4:**
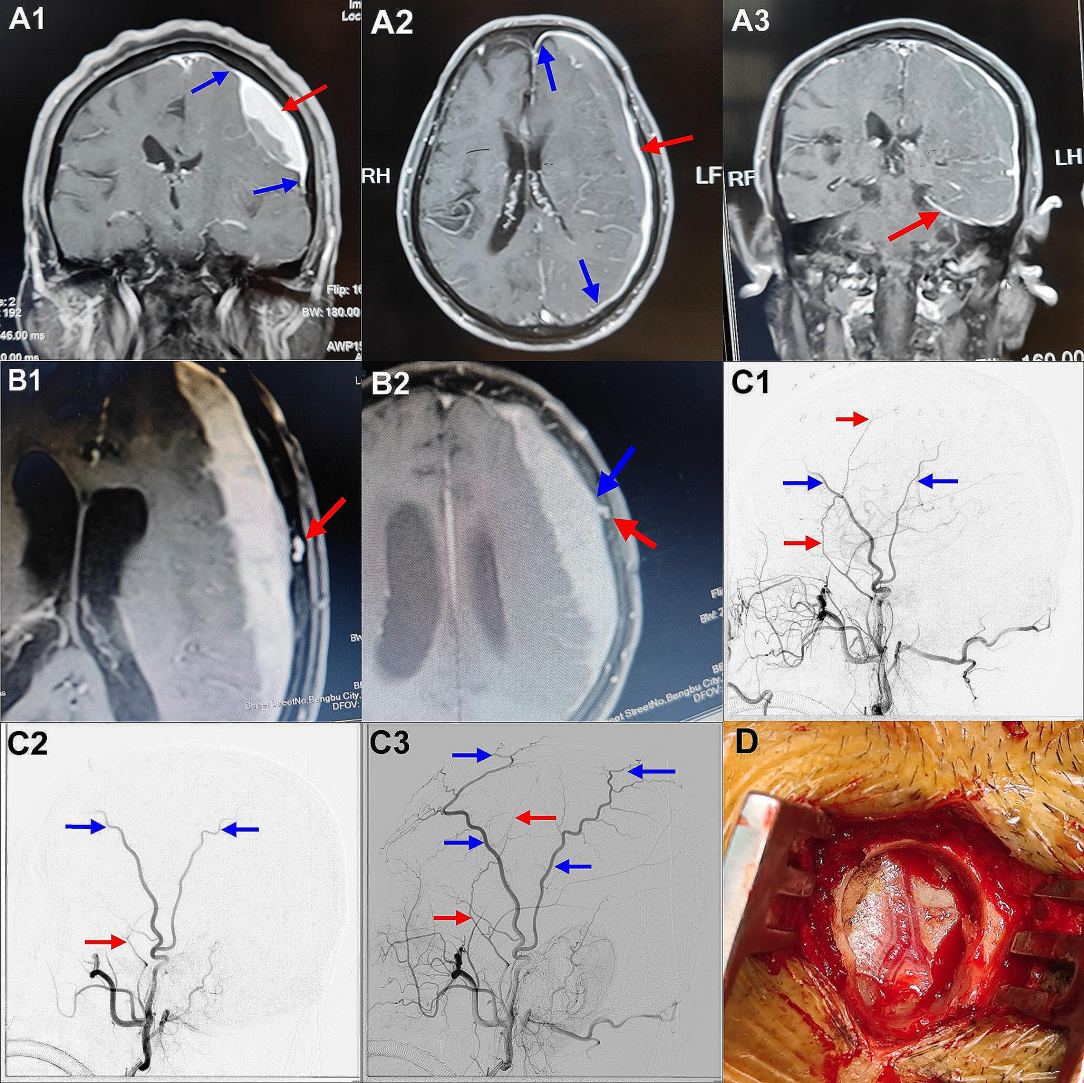
4A1 Magnetic resonance enhanced coronal view showing dural enhancement (red arrow) beyond the hematoma and the “dural tail sign” (blue arrow). 4A2 MRI enhanced level shows dural enhancement on the hematoma side (red arrow) beyond the hematoma and the “dural tail sign” (blue arrow). 4A3 Magnetic resonance enhanced coronal view shows both dural enhancement on the side of the hematoma and enhancement of the tentorium cerebelli (red arrow) away from the hematoma. 4B1 A patient’s MRI enhanced horizontal view shows a space between the MMA (red arrow) and the base of the hematoma. 4B2 Enhanced MRI horizontal view of the same patient showing a vascular connection between the MMA (red arrow) and the top of the hematoma (blue arrow). 4C1 Preoperative lateral imaging of the external carotid artery in a patient shows that the MMA (red arrow) was developed before the STA (blue arrow). 4C2 In this patient, the main MMA stem could not be accurately located during the operation, hence, only the small branches of the MMA were identified and severed, and the development of the MMA (red arrow) was slower than that of the STA (blue arrow) after surgery. 4C3 The complete MMA (red arrow) and STA (blue arrow) development of this patient after surgery is different from Fig. 4C1, showing that STA is fully developed, while MMA is not fully developed. 4D Intraoperative MMA was found to be relatively thick

**Fig. 5:**
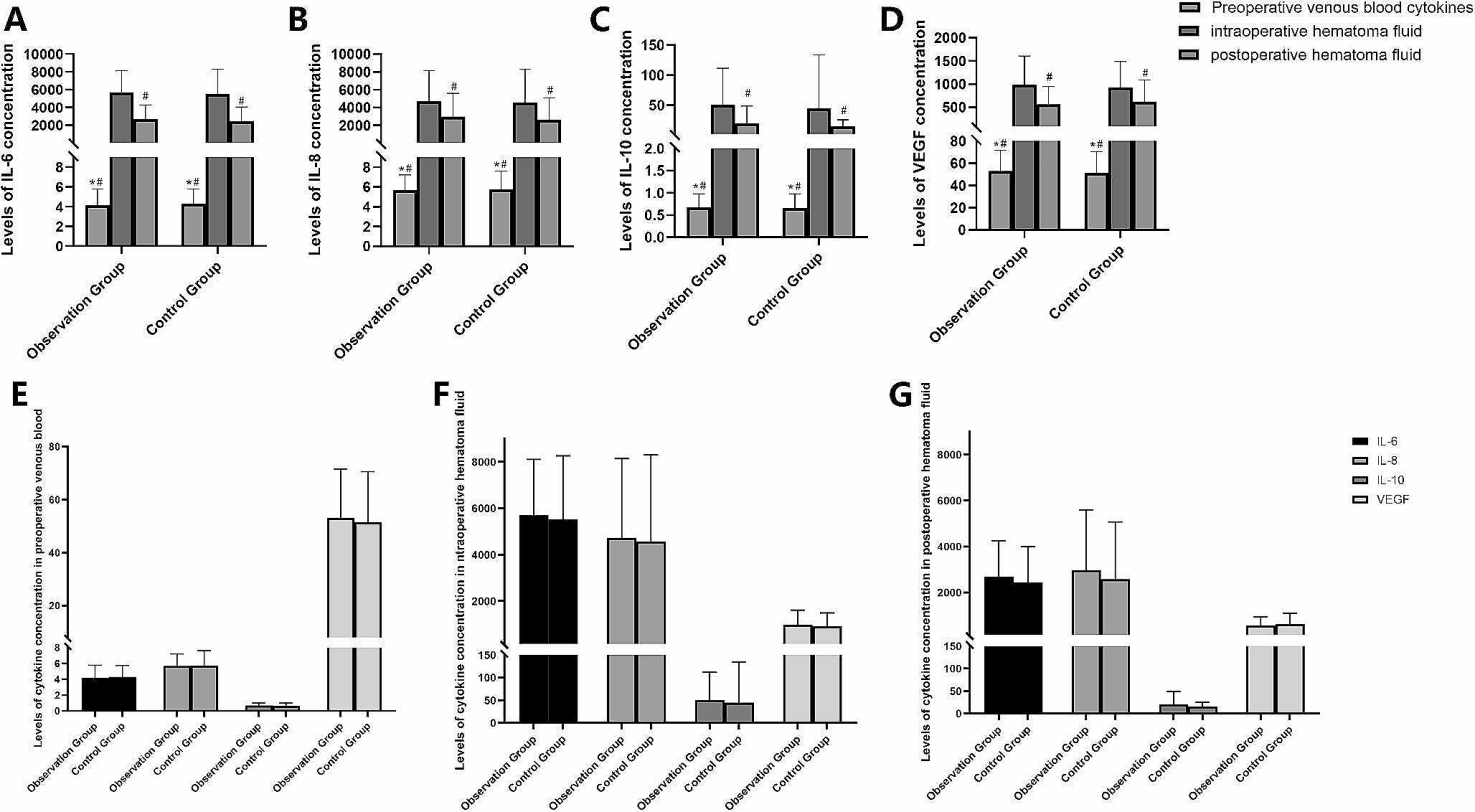
Concentrations of cytokines in preoperative venous blood and in intraoperative and postoperative hematoma fluid (pg/mL) * Compared with postoperative hematoma fluid, *P* < .05 # Compared with intraoperative hematoma fluid, *P* < .05

### MRS score

The average mRS scores of the observation group and the control group preoperatively were 3.19 ± 0.92 and 3.11 ± 1.01, respectively. The average mRS scores of the observation group and the control group before discharge were 0.33 ± 0.48 and 0.38 ± 0.68, respectively. There were no statistically significant differences in the mRS scores between the two groups pre- and postoperatively. There were statistically significant differences in the mRS scores between the two groups pre- and postoperatively (Table 2).

**Table 2:** Pre- and postoperative mRS scores in the observation and control groups

mRS score (mean ± SD)	Observation Group(*N* = 27)	Control Group(*N* = 45)	*P*-value
pre-operation	3.19 ± 0.92	3.11 ± 1.01	0.79
post-operation	0.33 ± 0.48	0.38 ± 0.68	0.85
*P*-value	<0.05	<0.05	

### Preoperative and intraoperative complications

There were no postoperative complications, such as infections, rebleeding, cerebrospinal fluid leakage, brain contusion or laceration, seizures, or poor healing of scalp incisions, in the two groups.

### Catheter extubation time

The average indwelling time of the drainage tube on 29 sides in 27 patients in the observation group was 2.04 ± 0.61 days, while the average indwelling time of the drainage tube on 48 sides in the 45 patients in the control group was 2.48 ± 0.61 days. The differences in catheter remaining time between the two groups were statistically significant (Table 3).

**Table 3:** Drainage tube retention time and recurrence rate in the observation and control groups

	Observation Group (*N* = 29)	Control Group (*N* = 48)	*P*-value
Drainage tube retention time	2.04 ± 0.61	2.48 ± 0.61	<0.05
Recurrence rate	0	4	<0.05

**Table 4:** DSA on hematoma side and non-hematoma side showed STA and MMA development sequence in observation group

	Hematoma side (*N* = 29)	Non-hematoma side (*N* = 25)	*P*-value
STA preceded MMA	14/29(48.3%)	20/25(80.0%)	0.016^a^
Simultaneous occurrence of STA and MMA	4/29(13.8%)	4/25(16.0%)	1.00
STA appears after MMA	11/29(37.9%)	1/25(4.0%)	<0.05

### Recurrence rate

Patients in both groups were followed up for 5 months. There was no recurrence of hematomas on 29 sides in the 27 patients in the observation group; however, the hematoma recurred on four sides out of 48 sides in the 45 patients in the control group, and required surgical re-intervention. The recurrence rate was 8.33%, and the difference in the recurrence rates between the two groups was statistically significant (Table 3).

### Imaging results

1. External carotid artery DSA was performed on 27 patients with hematomas on 29 sides in the observation group. The contrast agent was used in the arterial, capillary, and venous phases until the contrast agent disappeared, and no “cotton wool sign” was seen.
2. Blood flow velocity: DSA of the external carotid artery on 29 sides in 27 patients with hematomas in the observation group showed that the MMA on 11 sides had developed before the STA; on four sides, the MMA and the STA developed almost simultaneously; and on 14 sides, the STA developed before the MMA. DSA of the external carotid artery on 25 sides in 27 patients without hematomas showed that on 20 sides, the STA was developed before the MMA; on four sides, the MMA and STA developed almost simultaneously; and on one side, the MMA was developed before the STA. There was a statistically significant difference between the patients in whom the MMA developed before the STA as well as the healthy side in the observation group [Table 1]. After incising the MMA, DSA showed that in cases where the preoperative MMA developed before or simultaneously with the STA, it developed slower than the STA postoperatively [Fig. 3A1A2].

3. DSA of the external carotid artery combined with the location of the upper and lower boundaries of the hematoma in the observation group showed that the hematoma was mainly concentrated in the distribution range of the anterior branch of the MMA, and the anterior branch of the MMA was thicker than the posterior branch in the distribution area of the hematoma. Furthermore, the posterior branch of the MMA in most patients was distributed at the lower boundary of the hematoma, or a small number of thin posterior branches were distributed in the range of the hematoma. [Figure 3B1B2]
4. The location of the MMA puncture point identified using preoperative DSA in the observation group was found that the position of MMA blood supply interrupted was approximately 1.5 cm lower than the puncture point after the operation. [Figure 3C1C2]
5. Preoperative DSA of the external carotid artery in the observation group showed that the diameter of the MMA on the lesion side of the same patient at the same level and height was slightly thicker than that on the healthy side. Seven patients in the control group underwent head MRA imaging, which showed that the MMA on the affected side was more obvious and thicker under the hematoma, while the MMA on the healthy side was often unclear or small [Fig. 3D1D2].
6. Twenty-two patients in the two groups underwent head MR examinations, which showed that the dural enhancement on the affected side and part of the dura formed a thick enhancement layer, and the edge of the enhanced dura often exceeded the hematoma, similar to the “dural tail sign” of meningiomas [Fig. 4A1A2]. A few other patients also had enhancement of all the dural tissue on the affected side, including the tentorium. There was no enhancement of the hematoma and the medial wall of the hematoma [Fig. 4A3], and there was no enhancement of the dura on the non-hematoma side. In the enhanced MR images, the enhanced MMA seemed to have a closer relationship with the dura at the distal end of the hematoma and had vascular connections, while the MMA had no obvious contact at the proximal and middle ends of the hematoma [Fig. 4B1B2].

## Discussion

20 years ago, Mandai et al. [29] reported for the first time that a patient with refractory CSDH with recurrent recurrence was successfully cured by embolization with MMA. Since then, numerous neurosurgeons have adopted MMA embolization as a new method for treating CSDH [30–32]. In February 2024, the preliminary results of the EMBOLISE (Embolization of the MMA with Onyx liquid embolic system in the treatment of subacute and chronic subdural hematoma), STEM(SQUID trial for the embolization of the MMA for treatment of chronic subdural hematoma) and MAGIC-MT (Managing non-acute subdural hematoma using liquid materials: a Chinese randomized trial of MMA treatment) trial were presented at the International Stroke Conference. All three trials achieved their primary efficacy endpoint, demonstrating the effectiveness of MMAE. By embolizing the MMA, the source of blood for neovascularization is blocked, which cuts off the source of hematoma formation, thereby preventing the progression and recurrence of CSDH [26, 33]. After a period of absorption, the hematoma eventually dissipates [34]. However, MMA embolization has several limitations. First, in addition to angiogenic factors, CSDH also has inflammatory stimulating factors [35]. If only the angiogenic factors are removed, the inflammatory stimulating factors in the hematoma fluid will facilitate the recurrence and progression of the disease, which results in recurrence after MMA embolization [36–38]. A combined surgical approach may be the most beneficial treatment option for such patients. A systematic review reported that the recurrence rate of combined treatment is lower than that of evacuation surgery alone. The study also showed that combined treatment has a lower recurrence rate compared with that of evacuation surgery alone [39]. Additionally, the most prominent problem with simple MMA embolization is that the median time for hematoma regression after surgery is 5 months [40], which contradicts the original intention of patients who seek medical treatment for urgent relief of symptoms. A clinical survey from 90 countries showed that, unless there are specific indications, most patients with CSDH are unwilling to undergo MMA embolization [41]. Patients desire prompt recovery and relief from the pain of the disease. Additionally, embolization is associated with complications [39, 42], If interventional surgery can be avoided, minimally invasive drilling surgery utilizing a hole in the cranium with a diameter of several millimeters to occlude the MMA presents a viable option. This approach not only removes the angiogenic factors involved in the pathogenesis of the disease but also facilitates the discharge of the hematoma and inflammatory stimulating factors.

External carotid artery angiography of the observation group revealed that the number of cases in which the MMA was developed before the STA on the side with the hematoma was significantly more than those in which it occurred on the non-hematoma side, and the differences were statistically significant. After the main MMA was resected intraoperatively, the following part and collaterals of the blocked MMA were found to have a significantly slower development speed than that before the operation. Moreover, in cases where MMA could not be accurately located that were excluded, it was found that even if the main trunk of MMA was not severed and only the small branches of MMA were severed, there would still be a phenomenon of slow development of MMA compared to STA after surgery[Figure 4C1,C2,C3]. In addition to the change in blood flow velocity, the main trunk of the MMA on the hematoma side was also thicker than that on the healthy side, which can be confirmed in this study [Fig. 2A2, Fig. 3D2, Fig. 4D] and some literature [43, 44]. This is a very interesting finding, which can be explained by referring to the CT perfusion principle. Fast blood flow velocity means fast peak time, and thicker MMA means increased blood volume of the vessel, which indicates that the blood flow of the MMA on the hematoma side is increased compared with that on the non-hematoma side. Whether the increased blood flow and thickened MMA diameter on the lesion side are caused by local increased metabolism or local pathological changes needs to be studied further. The patient’s MR enhancements showed that the visceral layer (inner layer) of the hematoma in cases of CSDH was not enhanced. Similarly, the parietal layer (outer layer) of the hematoma also seemed not to be enhanced, and only the dura mater was enhanced because the enhanced dura mater exceeded the edge of the hematoma, and the thickness of the enhanced dura mater was the same at the site of the hematoma and outside the hematoma. In some cases, the whole dura mater was enhanced, which may be due to thickening of the vessels in the meninges on the affected side and an increase in blood flow velocity, resulting in increased blood flow throughout the dura mater.

The preoperative coagulation-related function tests of the two groups of patients, such as platelets, PT, APTT, D-Dimer, and venous blood cytokines, including the levels of IL6/8/10, VEGF, and CRP, were all within normal ranges; therefore, it can be speculated that CSDH is not a systemic coagulation disease or inflammatory disease. Analysis of the hematoma fluid collected intraoperatively revealed that the concentrations of IL6/8/10 and VEGF were abnormally increased, indicating that CSDH was a local inflammatory and angiogenic disease. After repeated washing of the hematoma cavity with saline, a drainage tube was placed for continuous drainage. The concentrations of IL6/8/10 and VEGF in the drainage fluid before extubation were still high; however, compared with the levels in the hematoma fluid obtained without saline washing, they had been greatly reduced, and the comparison of the two values was statistically significant. This indicates that active washing intraoperatively can reduce the concentrations of liquid inflammatory cytokines and vascular cytokines in the hematoma cavity, which facilitates recovery. This also explains why using saline to wash the hematoma cavity during surgery can reduce the recurrence rate of CSDH. Furthermore, some studies reported that if the hematoma cavity is washed with saline at a higher temperature, the recurrence rate can be reduced even further, because the solubility of organic solvents doubles with every 20 °C increase in temperature [45].

There were no complications, such as infection, rebleeding, or epilepsy, in both groups postoperatively. The postoperative MRS scores were also greatly improved, indicating that the surgery was relatively safe. Statistical comparisons indicated that the average drainage tube indwelling time in the observation group was less than that in the control group, which is beneficial to patients. Kim [46] explained this phenomenon by examining the higher brain reduction rate in patients with refractory CSDH who were treated with MMA embolization. After the MMA is embolized, the formation of a new hematoma membrane, vascular hyperpermeability, and inflammatory exudation can all be reduced; hence, the vicious cycle of recurrent bleeding can be broken as the formation of hematomas is reduced, absorption is accelerated, and reduction in brain volume becomes easier.

The recurrence rate in the observation group was lower than that in the control group, which can be theoretically explained by the pathogenesis of CSDH. Since CSDH is believed to be supplied by the dural branch of the MMA, surgical disconnection of the MMA can block its blood supply, thereby eliminating the processes of recurrent bleeding and chronic leakage [26]. Additionally, through drainage surgery to discharge the hematoma and by repeatedly rinsing the hematoma cavity with saline, inflammatory pathogenic factors in the hematoma cavity are cleared and reduced. Similarly, angiogenic factors and inflammatory mediators are removed to eliminate the pathophysiological basis of hematoma formation, and over time, the brain gradually absorbs the accumulated blood in the subdural space, thus reducing the recurrence rate.

### Limitations

This study has some limitations. First, the sample size of the study was small, especially the number of patients in the observation group; therefore, further accumulation of clinical data and validation of this study’s findings are needed. Second, the types of inflammatory and vascular cytokines in the hematoma fluid were few, and the level of cytokines in the hematoma fluid before extubation was relatively large. This may be related to the non-standardization of the time and number of intraoperative saline washes. Third, because only a bone hole with a diameter of approximately 8 mm is drilled on the skull during this operation, the exposure of the dura mater is small; hence, the number of dura mater specimens was small and could not be used for extensive pathological evaluation of the diseased dura mater. Fourth, this study did not evaluate the blood flow parameters of the external carotid artery in detail. In future studies, ultrasound or image perfusion methods can be considered for specific numerical measurements to provide strong support for the hemodynamic changes of CSDH. Therefore, although this study is in its early stage, it contributes to broader research endeavors aimed at developing and exploring new treatment methods for CSDH. Further research in the later stages would need to increase the sample size, collect more data, and find a surgical method for locating the MMA without angiography, to validate our findings while also being more beneficial to patients with CSDH.

## Conclusion

External drainage of the drill hole in patients with CSDH can reduce the levels of IL6/8/10 and VEGF, as well as other cytokines in the hematoma fluid. Resecting the MMA on the dura during external drainage can reduce the recurrence rate of CSDH and reduce the drainage tube indwelling time. This approach is as simple and easy as traditional external drainage procedures; in addition, it has fewer complications and achieves greater improvement in neurological function.

## Data Availability

No datasets were generated or analysed during the current study.

## Abbreviations

CSDH: Chronic subdural hematoma
CT: Computed Tomography
MR: Magnetic Resonance
DSA: Digital Subtraction Angiography
MMA: Middle Meningeal Artery
